# Molecular detection of *Leishmania* spp. in feline squamous cell carcinoma in Portugal

**DOI:** 10.3389/fvets.2026.1952747

**Published:** 2026-09-04

**Authors:** Carolina Oliveira, Joana Santos, Andreia Valença, José Catarino, David Ramilo, Martim Matias, Margarida Alves, Carla Maia, Pedro Faísca, Joaquim Henriques, André Pereira

**Affiliations:** 1 Faculty of Veterinary Medicine, Lusófona University, Lisbon University Centre, Lisbon, Portugal; 2 Research in Veterinary Medicine (I-MVET), Faculty of Veterinary Medicine, Lusófona University, Lisbon University Centre, Lisbon, Portugal; 3 DNAtech Veterinary Laboratory, Lisbon, Portugal; 4 Superior School of Health, Protection and Animal Welfare (ESPA), Polytechnic Institute of Lusophony (IPLUSO), Lisbon, Portugal; 5 Veterinary and Animal Research Centre (CECAV), Faculty of Veterinary Medicine, Lusófona University, Lisbon University Centre, Lisbon, Portugal; 6 Research Center for Biosciences and Health Technologies (CBIOS), Lisbon University Centre, Lusófona University, Lisbon, Portugal; 7 Global Health and Tropical Medicine (GHTM), LA-REAL, Instituto de Higiene e Medicina Tropical, Universidade Nova de Lisboa, Lisbon, Portugal; 8 iNOVA4Health, NOVA Medical School, Universidade Nova de Lisboa, Lisbon, Portugal; 9 Anicura Atlântico Hospital Veterinário, Mafra, Portugal

**Keywords:** cats, feline leishmaniosis, formalin-fixed paraffin-embedded tissue, molecular detection, Portugal, squamous cell carcinoma

## Abstract

Cutaneous manifestations of feline leishmaniosis may occur at anatomical sites commonly affected by squamous cell carcinoma (SCC), creating potential diagnostic overlap. Although concurrent *Leishmania* infection and SCC have been described in individual cats, the presence of parasite DNA in feline SCC tissue has not been systematically investigated. This retrospective cross-sectional study evaluated 219 formalin-fixed, paraffin-embedded feline SCC samples submitted to a veterinary diagnostic laboratory in Portugal between 2020 and 2023. Samples were screened using a *Leishmania*-genus-specific nested PCR targeting SSU rDNA. PCR-positive cases were subsequently analyzed at four additional loci (*cytB*, *g6pdh*, *hsp70*, and ITS rDNA) and reassessed histopathologically. *Leishmania* spp. SSU rDNA was detected in 3/219 samples (1.4%; 95% CI: 0.5–3.9%), corresponding to SCC lesions on the pinna, nasal planum, and eyelid. Molecular characterization was possible in one case, involving an SCC of the nasal planum. Phylogenetic analysis showed that the *hsp70* sequence clustered with reference sequences of *L. infantum*, whereas the *cytB* sequence grouped within the *L. donovani* complex without species-level resolution. In the same lesion, structures morphologically compatible with amastigotes were observed within macrophages in the tumor-associated inflammatory infiltrate. Overall, *Leishmania* DNA was detected in only a small proportion of feline SCCs, and the findings support coexistence rather than an association between infection and tumor development. These findings have direct diagnostic relevance in endemic areas, where histopathologic confirmation of SCC should not preclude investigation for concurrent *Leishmania* infection when clinical or epidemiologic suspicion persists.

## 1 Introduction

Feline leishmaniosis (FeL), predominantly caused by *Leishmania infantum*, is increasingly recognized in endemic regions but remains considerably less well characterized than canine leishmaniosis (1–3). In Portugal, a recent nationwide serosurvey reported an overall seroprevalence of 8.9% in cats (4). Clinical disease is most often characterized by cutaneous lesions, including ulcerative, crusting, exfoliative, and nodular dermatitis, predominantly affecting the head and distal limbs (1, 5). Systemic manifestations may also occur, particularly in cats with concurrent conditions that may compromise immune function, including feline immunodeficiency virus infection, feline leukemia virus infection, and neoplasia (1, 6).

*Leishmania* DNA has been detected in several feline tissues and biological samples, including skin, lymph nodes, spleen, blood, bone marrow, and mucosal samples (1, 7–9). Amastigotes have also been demonstrated in some tissues by cytology or histopathology (1, 10). Nevertheless, the tissue distribution of the parasite and its clinical significance in cats remain incompletely understood, and current knowledge is still largely derived from individual cases and extrapolation from canine leishmaniosis (2, 6).

Squamous cell carcinoma (SCC) is among the most common cutaneous malignancies in cats and typically develops in sparsely haired, lightly pigmented, and ultraviolet-exposed areas, particularly the nasal planum, pinnae, and eyelids (11). These anatomical sites overlap with the distribution of many FeL-associated cutaneous lesions, creating potential diagnostic ambiguity. Cats presenting with ulcerative or nodular lesions initially suspected to represent SCC have subsequently been diagnosed with leishmaniosis in the absence of neoplasia (12). Conversely, the coexistence of *Leishmania* infection and SCC within the same lesion has been reported in individual cats (13–16).

The relationship between *Leishmania* infection and neoplasia has also received attention in human medicine. Leishmaniosis may mimic neoplastic disease, including SCC, and parasite infection and malignancy have occasionally been documented within the same tissue (17, 18). Although chronic inflammation and parasite-mediated modulation of the host immune response have been proposed as potential mechanisms linking *Leishmania* infection and tumorigenesis (19), epidemiological evidence supporting a causal relationship remains lacking, and the available evidence is largely based on case reports and clinical observations (20).

Despite these observations, the occurrence of *Leishmania* DNA in feline SCC tissue has not been systematically investigated. Based on the hypothesis that *Leishmania* DNA is present in feline SCC tissue from cats in Portugal, this study aimed to estimate its detection frequency and, in positive cases, to characterize the parasite and assess the associated histopathologic findings.

## 2 Materials and methods

### 2.1 Study design and sampling

This retrospective cross-sectional study considered all archived formalin-fixed, paraffin-embedded (FFPE) tissue samples with a histopathologic diagnosis of squamous cell carcinoma (SCC) submitted for routine diagnostic evaluation between 2020 and 2023 to a veterinary diagnostic laboratory in Portugal (DNAtech, Lisbon). FFPE blocks with insufficient residual tissue for molecular analysis were excluded, and only one SCC sample per cat was considered eligible. The resulting sampling frame comprised 392 cases, from which 219 were selected by simple random sampling. The required sample size was estimated using EpiTools, assuming an expected molecular detection frequency of 5.4% (21), a precision of 3% and a 95% confidence level.

Information retrieved from the original submission records included breed, sex, age, geographical origin (classified according to the Nomenclature of Territorial Units for Statistics—NUTS II) and tumor location. Anatomical location was recorded as stated in the submission form. Cases that could not be assigned to the predefined categories of ear pinna, eyelid, nasal planum, or oral cavity were grouped as “other or unspecified sites.” Age groups were defined as young (1–6 years), mature (7–10 years), and senior (> 10 years) (4). The study did not include non-neoplastic control tissues.

### 2.2 DNA extraction

Three to five serial sections, 10 μm thick, were obtained from each FFPE block, depending on tissue availability. The microtome blade was replaced between blocks, and the microtome was decontaminated with 1.0% active sodium hypochlorite and subsequently rinsed with sterile distilled water to remove residual disinfectant. Sections were deparaffinized with xylene and ethanol washes, and DNA was extracted using the innuPREP FFPE DNA Kit (IST Innuscreen, Berlin, and Germany) according to the manufacturer’s instructions. Extraction blank controls were included during DNA extraction. DNA concentration and spectrophotometric purity were assessed using a NanoDrop® 1,000 spectrophotometer (Thermo Scientific, United States). DNA integrity was qualitatively evaluated by electrophoresis on 0.8% agarose gels, and amplifiability was assessed by PCR amplification of a 177-bp fragment of the feline beta-glucuronidase gene (*GUSB*) (Supplementary Table S1).

### 2.3 Molecular detection and characterization of *Leishmania* spp.

Screening for *Leishmania* DNA was performed by nested PCR targeting the SSU rDNA (22). PCR reactions were performed using NZYTaq II 2 × Green Master Mix (NZYTech, Lisbon, Portugal; catalog no. MB35801). Amplification products were separated by electrophoresis on 1.5% agarose gels stained with GreenSafe Premium (NZYTech, Lisbon, Portugal; catalog no. MB13201) and visualized under ultraviolet illumination; fragment sizes were assessed by comparison with NZYDNA Ladder V, 100–1,000 bp (NZYTech; catalog no. MB06103). Samples testing positive for *Leishmania* SSU rDNA were subsequently analyzed at four loci: cytochrome b (*cytB*), glucose-6-phosphate dehydrogenase (*g6pdh*), heat-shock protein 70 (*hsp70*) and the internal transcribed spacer region of the ribosomal DNA (ITS rDNA), using previously described protocols (21) (Supplementary Table S1). All molecular assays were performed directly on the original DNA extracts from the FFPE SCC samples. Template input was standardized by volume rather than DNA concentration, with 10 μL of DNA extract used for the first round of the SSU rDNA nested PCR and 5 μL for the remaining PCR assays, as detailed in Supplementary Table S1. Positive controls consisting of genomic DNA from *L. infantum* (MON-1; MHOM/PT/88/IMT318), and no-template controls containing nuclease-free, ultrapure water instead of template DNA, were included in each PCR run.

### 2.4 Sequencing and phylogenetic analysis

PCR products were purified using the innuPREP PCRpure Kit (IST Innuscreen) and sequenced in both directions by the Sanger method at STABVIDA (Caparica, Portugal), using the same primers employed for PCR amplification. Nucleotide sequence identity was assessed using BLASTn against the NCBI nucleotide database. Consensus sequences were aligned with homologous sequences retrieved from the NCBI GenBank database using the iterative G-INS-i refinement method implemented in MAFFT v7. Alignments were processed with Gblocks (23) via SeaView v5.0.5. Protein-coding alignments were subsequently inspected in AliView v1.28 to preserve the correct reading frame.

Maximum-likelihood phylogenetic analyses were performed in IQ-TREE v2.4.0 (24), with the best-fitting nucleotide substitution model selected according to the Bayesian Information Criterion (25). Branch support was assessed by bootstrap resampling with 1,000 standard replicates, and values ≥ 75 were considered indicative of robust topological support. *Leishmania tarentolae* was used as the outgroup, and the resulting trees were visualized and edited in FigTree v1.4.4. Sequences generated in this study were deposited in the DDBJ/ENA/GenBank databases under accession numbers LC919850 and LC919851.

### 2.5 Histopathology

The FFPE blocks corresponding to SCC samples in which *Leishmania* SSU rDNA was detected were re-sectioned for histopathologic examination. Sections were stained with hematoxylin and eosin and digitized using a slide scanner (NanoZoomer SQ; Hamamatsu Photonics, Shizuoka, Japan). Digital slides were reviewed to confirm tumor morphology and to identify parasitic structures and associated inflammatory or tissue changes.

### 2.6 Statistical analysis

Descriptive statistical analysis was performed using SPSS Statistics v25 (IBM Corp., Armonk, NY, United States). The frequency of *Leishmania* DNA detection was calculated as the proportion of PCR-positive samples among all SCC samples tested, and corresponding 95% confidence intervals (CI) were calculated using Wilson’s method.

## 3 Results

DNA concentrations ranged from 11.4 to 440 ng/μL (median, 48.6 ng/μL), and A260/A280 ratios ranged from 0.9 to 2.0 (median, 1.7). All 219 DNA extracts yielded the expected 177-bp feline *GUSB* amplicon.

*Leishmania* SSU rDNA was detected in three of the 219 samples (1.4%; 95% CI: 0.5–3.9%). All three positive samples originated from domestic shorthair/European Shorthair cats with SCC located on the head, namely the ear pinna, nasal planum, and eyelid. These cats were from three different NUTS II regions (Norte, Centro, and Oeste e Vale do Tejo; Table 1); one was one year old, and another was ten years old; age was not available in the submission records for the cat whose SCC was located on the eyelid. Detection frequencies according to age group, sex, breed, NUTS II region, and tumor location are described in Table 1.

**Table 1:** Molecular frequency of *Leishmania* spp. in feline squamous cell carcinoma according to age, sex, breed, region and tumor location.

Variable/categories	Positive/tested	Frequency, % (95% CI)
Age group	186	
Young	1/36	2.8 (0.5–14.2)
Mature	1/74	1.4 (0.2–7.3)
Senior	0/76	0.0 (0.0–4.8)
Sex	219	
Female	3/106	2.8 (1.0–8.0)
Male	0/113	0.0 (0.0–3.3)
Breed	219	
Domestic shorthair/European Shorthair	3/198	1.5 (0.5–4.4)
Maine Coon	0/1	0.0 (0.0–79.3)
Norwegian Forest Cat	0/1	0.0 (0.0–79.3)
Persian	0/9	0.0 (0.0–29.9)
Siamese	0/10	0.0 (0.0–27.8)
NUTS region	219	
Norte	1/20	5.0 (0.9–23.6)
Centro	1/39	2.6 (0.5–13.2)
Oeste e Vale do Tejo	1/23	4.3 (0.8–21.0)
Grande Lisboa	0/50	0.0 (0.0–7.1)
Península de Setúbal	0/36	0.0 (0.0–9.6)
Alentejo	0/8	0.0 (0.0–32.4)
Algarve	0/34	0.0 (0.0–10.2)
Açores	0/8	0.0 (0.0–32.4)
Madeira	0/1	0.0 (0.0–79.3)
Tumor location	219	
Ear pinna	1/59	1.7 (0.3–9.0)
Eyelid	1/16	6.3 (1.1–28.3)
Nasal planum	1/51	2.0 (0.3–10.3)
Oral cavity	0/49	0.0 (0.0–7.3)
Others or unspecified	0/44	0.0 (0.0–8.0)
Overall	3/219	1.4 (0.5–3.9)

CI, Confidence interval; NUTS, Nomenclature of territorial units for statistics.

Among the SSU rDNA-positive samples, only the sample from the SCC located on the nasal planum yielded amplification products suitable for sequencing, at the *hsp70* and *cytB* loci. No amplification products were detected for *g6pdh* or ITS rDNA in any sample, and none of the four characterization loci was amplified in the remaining two samples. BLASTn analysis showed 100% query coverage and 100% nucleotide identity between the *cytB* sequence and *L. infantum* reference sequences (e.g., GenBank accession no. LC460177), whereas the *hsp70* sequence showed 100% query coverage and 98% nucleotide identity with *L. infantum* reference sequences (e.g., GenBank accession no. OR136917).

**Figure 1:**
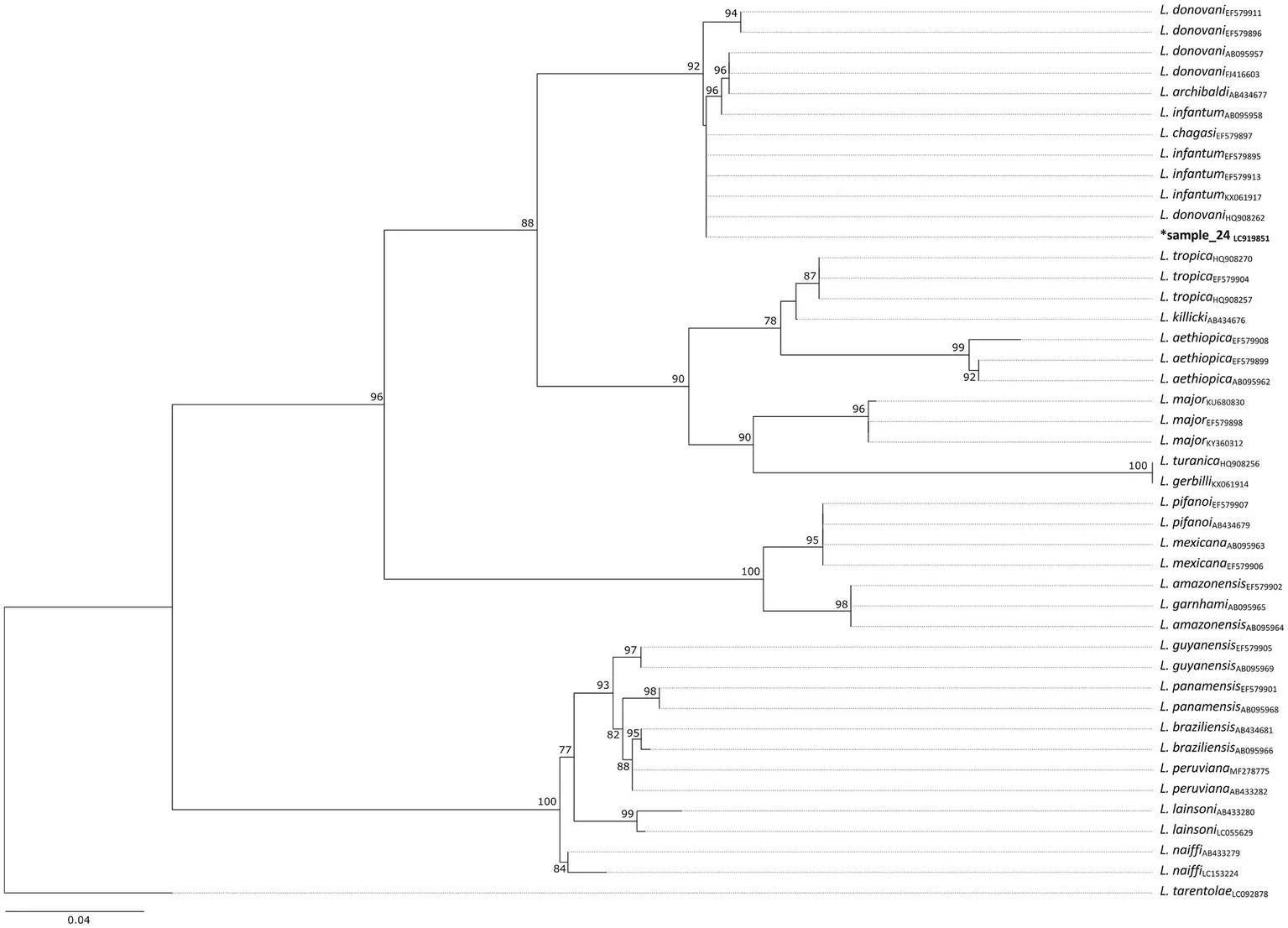
Maximum likelihood phylogenetic tree inferred from *hsp70* sequences of *Leishmania* spp. detected in squamous cell carcinoma in cats from Portugal. Tree reconstruction was performed using IQ-TREE under the TN + F + I nucleotide substitution model, selected according to the Bayesian Information Criterion. Node support was assessed using 1,000 bootstrap replicates, and values ≥75% are shown at the corresponding nodes. Reference sequences are labeled with species designation and GenBank accession numbers. The sequence obtained in this study is shown in bold, marked with an asterisk (*), and labeled with the sample ID and corresponding GenBank accession number. Branch lengths are scaled to the number of substitutions per site.

**Figure 2:**
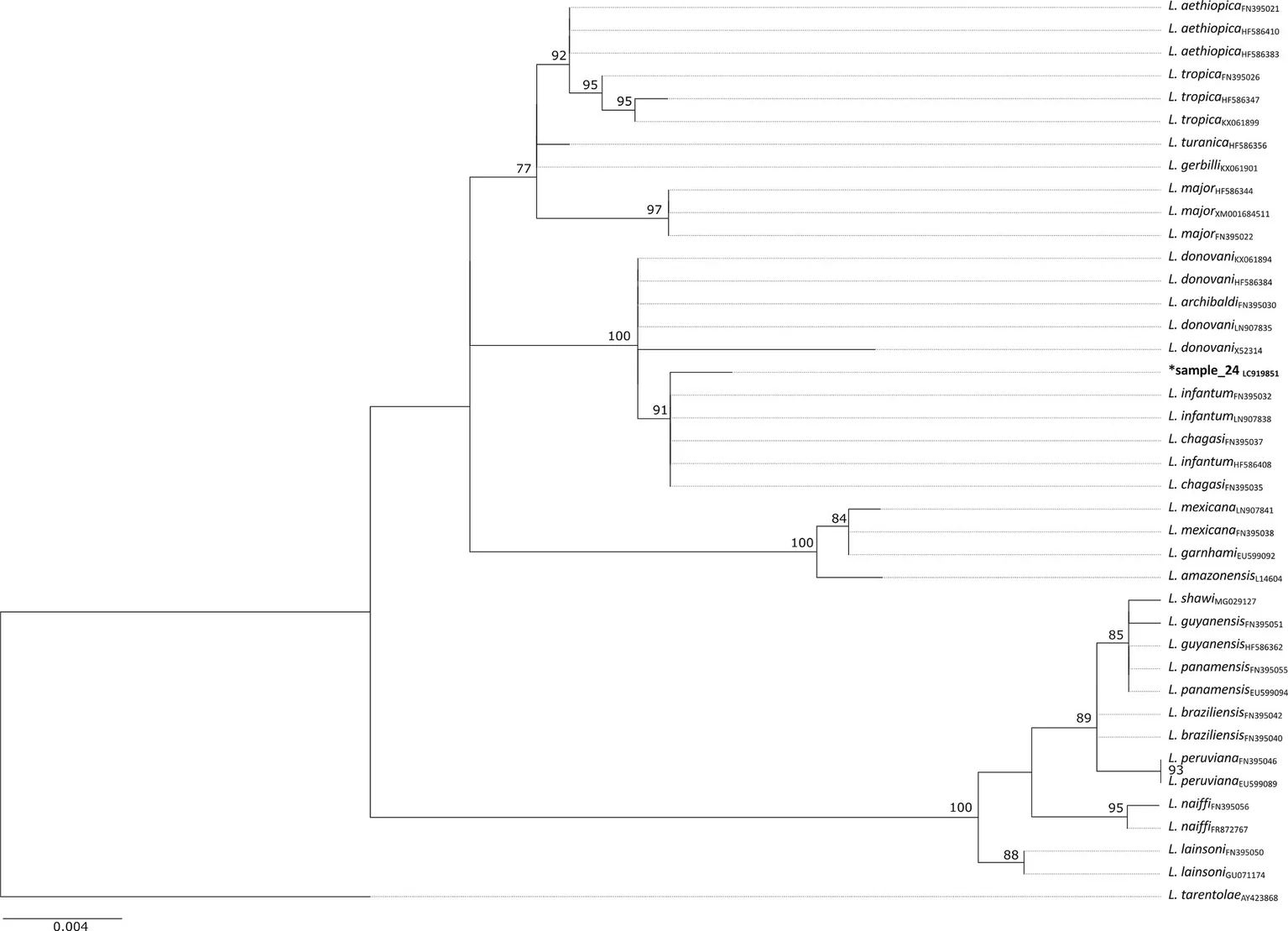
Maximum likelihood phylogenetic tree inferred from *cytB* sequences of *Leishmania* spp. detected in squamous cell carcinoma in cats from Portugal. Tree reconstruction was performed using IQ-TREE under the TN + F + G4 nucleotide substitution model, selected according to the Bayesian Information Criterion. Node support was assessed using 1,000 bootstrap replicates, and values ≥75% are shown at the corresponding nodes. Reference sequences are labeled with species designation and GenBank accession numbers. The sequence obtained in this study is shown in bold, marked with an asterisk (*), and labeled with the sample ID and corresponding GenBank accession number. Branch lengths are scaled to the number of substitutions per site.

Phylogenetic analysis revealed that the *hsp70* sequence clustered with reference sequences of *L. infantum* with 91% bootstrap support (Figure 1). The *cytB* sequence grouped within a clade containing reference sequences of *L. infantum* and *L. donovani*, supported by a bootstrap value of 92%, without species-level resolution (Figure 2).

**Figure 3:**
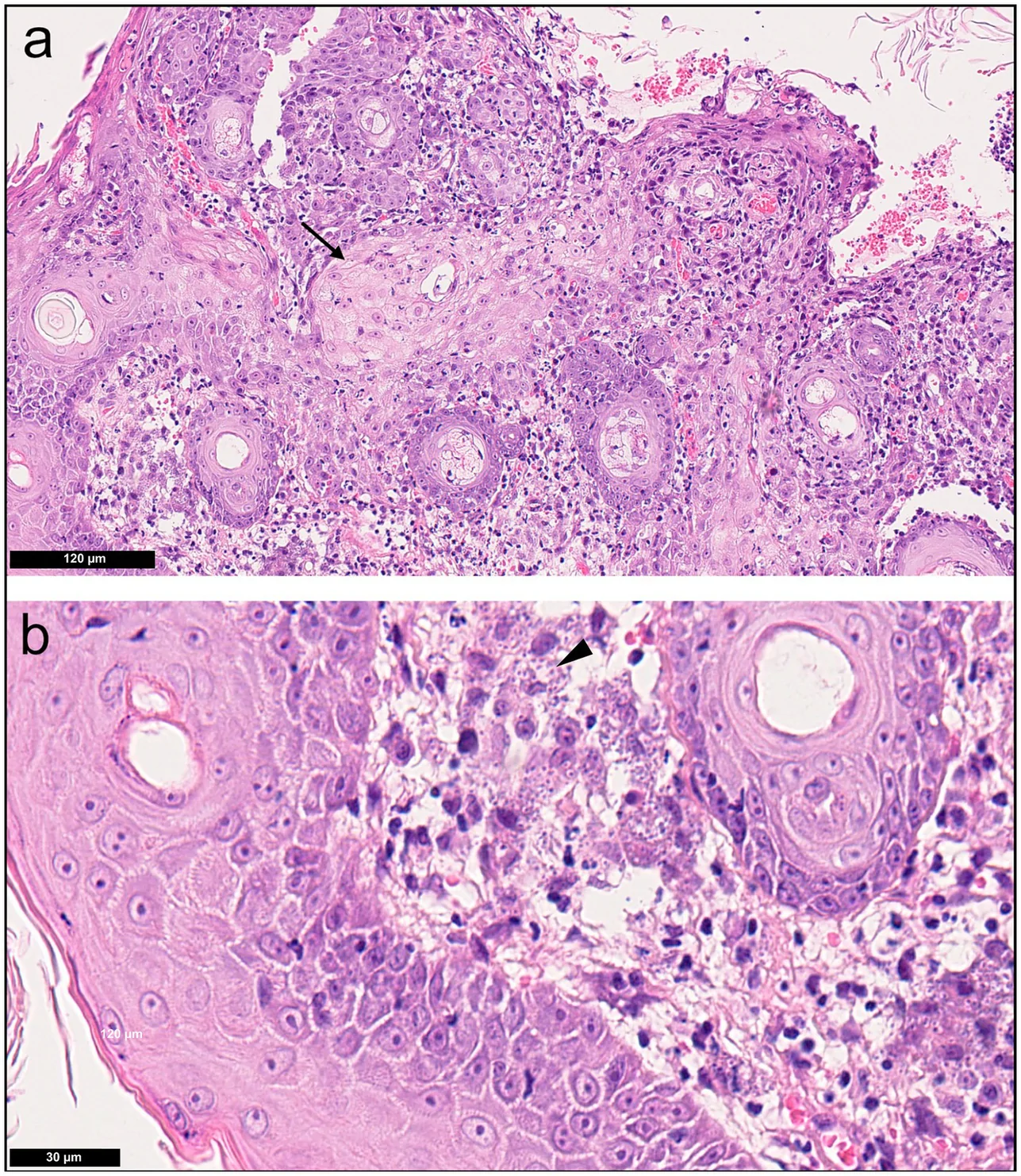
Histological images of the squamous cell carcinoma case in which structures morphologically compatible with amastigotes were microscopically detected. **(a)** Low magnification showing proliferating squamous epithelial cells arranged in trabeculae infiltrating the superficial dermis (arrow), accompanied by a moderate mixed inflammatory infiltrate. **(b)** Higher magnification highlighting macrophages, present in the superficial dermis, containing intracytoplasmic structures morphologically compatible with *Leishmania* spp. amastigotes (arrowhead). Hematoxylin and Eosin stain.

Histopathologic review confirmed SCC in all three PCR-positive cases. Structures morphologically compatible with *Leishmania* amastigotes were identified only in the nasal planum lesion, within the cytoplasm of macrophages in the dermal inflammatory infiltrate associated with the tumor (Figure 3).

## 4 Discussion

To our knowledge, this is the largest systematic molecular screening of feline neoplastic tissue for *Leishmania* spp. conducted to date and extends previous evidence based largely on individual case reports. Parasite DNA was detected in only 1.4% of the samples examined, indicating that its occurrence in feline SCC tissue is uncommon and does not support a frequent association between *Leishmania* infection and SCC. Nevertheless, the findings demonstrate that SCC and *Leishmania* infection can coexist within the same lesion.

The detection frequency observed in this study should be considered in the context of the type of material examined. Molecular detection frequencies reported in blood, conjunctival swabs, or fresh tissues from cats living in endemic areas are not directly comparable because sample type, molecular target, assay sensitivity, and template preservation differ substantially (7, 26, 27). Accordingly, direct comparison with molecular detection frequencies reported in other feline populations should be made cautiously.

All positive lesions occurred at anatomical sites on the head that are recognized predilection sites for feline cutaneous SCC and are also frequently affected by cutaneous manifestations of feline leishmaniosis (5, 11, 28). This anatomical overlap has direct diagnostic relevance. Feline leishmaniosis may clinically resemble SCC, particularly when presenting as nodular or ulcerative lesions of the nasal planum or pinnae (12). Conversely, concurrent SCC and *L. infantum* infection have been documented within the same lesion in individual cats (13–16). Nevertheless, lesion location alone is insufficient to infer infection, as *Leishmania* was not detected by immunohistochemistry or quantitative PCR in a previous series of cats with ulcerative dermatitis of the head and neck (29). Investigation for concurrent leishmaniosis should therefore be guided by the combined clinical, histopathologic, and epidemiologic context.

Molecular characterization provided species-level support for *L. infantum* through the *hsp70* analysis, whereas *cytB* did not discriminate between members of the *L. donovani* complex. Species assignment should therefore rely primarily on the *hsp70* result, together with the established circulation of *L. infantum* in Portugal (9, 15, 21, 30). The remaining PCR-positive samples could only be assigned to the genus level.

Structures morphologically compatible with amastigotes were observed only in the nasal planum lesion, despite *Leishmania* SSU rDNA detection in three samples. The absence of recognizable organisms in the remaining PCR-positive lesions may reflect a low or focal parasite burden, sampling variability between tissue sections, or the greater analytical sensitivity of PCR compared with microscopic detection (1, 7). In the nasal planum lesion, the histopathologic findings were concordant with the molecular detection and characterization of *Leishmania*. However, H&E morphology alone does not provide organism-specific confirmation, and *Leishmania*-specific immunohistochemistry would have provided additional validation of the observed intramacrophage structures (12).

In an endemic setting, incidental infection represents a plausible explanation for the detection of *Leishmania* DNA within SCC tissue. Alternatively, local persistence of the parasite within an inflamed or immunologically altered tumor microenvironment cannot be excluded. The findings therefore support coexistence but do not provide evidence that *Leishmania* infection contributes to SCC development or progression.

Clinically, these findings indicate that SCC and *Leishmania* infection may coexist in cats from endemic areas. Histopathologic confirmation of SCC should therefore not preclude investigation for concurrent leishmaniosis when compatible clinical findings or epidemiologic risk factors remain.

Recent work in another SCC setting has highlighted the value of assessing inflammatory and oncogenic biomarkers when investigating potential infection-associated changes within the tumor microenvironment (31). Future multicenter prospective studies involving larger cohorts and anatomically matched non-neoplastic controls should combine complementary molecular and tissue-based approaches. Quantitative PCR (qPCR) and droplet digital PCR (ddPCR) may improve parasite detection and enable estimation of parasite burden, particularly in samples containing low amounts of *Leishmania* DNA (27, 32). In parallel, *Leishmania*-specific immunohistochemistry and *in situ* hybridization can provide spatial confirmation of the parasite within tissue lesions and help characterize its distribution in relation to the inflammatory and neoplastic components (12, 33). Integration of inflammatory and oncogenic biomarkers could further characterize the local tumor microenvironment and its association with parasite presence.

Several limitations should be considered when interpreting these findings. First, the retrospective use of archival FFPE tissue imposes inherent constraints on molecular sensitivity, as formalin fixation and prolonged storage can fragment and chemically modify DNA, particularly affecting the amplification of longer targets (34, 35). Although all samples amplified the 177-bp feline *GUSB* fragment, this demonstrates only the presence of amplifiable short-fragment DNA and does not ensure preservation of the longer targets used for multilocus characterization. In addition, template input was standardized by volume rather than DNA concentration, and variation in DNA concentration and purity may therefore have affected PCR sensitivity. The absence of anatomically matched non-neoplastic controls precluded comparison of *Leishmania* detection frequency between neoplastic and non-neoplastic tissues, while the small number of PCR-positive cases limited broader interpretation. Finally, the lack of clinical information, retroviral status, hematologic data, treatment history, and follow-up precluded assessment of clinical leishmaniosis or concurrent immunosuppression.

## 5 Conclusion

*Leishmania* DNA was detected in a small proportion of feline SCC samples, expanding current epidemiological knowledge on the occurrence of the parasite in feline neoplastic tissue. The findings demonstrate that SCC and *Leishmania* infection can coexist within the same feline lesion but do not support a causal relationship between infection and tumor development. In endemic areas, histopathologic confirmation of SCC should therefore not preclude investigation for concurrent *Leishmania* infection when clinical or epidemiologic suspicion persists.

## Data Availability

The datasets generated and/or analyzed during the current study are available from the corresponding author on reasonable request. Sequences generated in this study were deposited in the DDBJ/ENA/GenBank databases under accession numbers LC919850 and LC919851.
