# Supplementary material for: Molecular detection of Leishmania spp. in feline squamous cell carcinoma in Portugal
Source: Front Vet Sci. 2026 Sep 4;13:1952747. doi: 10.3389/fvets.2026.1952747 (PMC13585706; doi:10.3389/fvets.2026.1952747)
Supplement: Supplementary file 1 [file Table_1.DOCX]

**Supplementary Table 1.** Molecular targets, primer sequences and PCR conditions used in this study.

| **Target organism** | **Target gene** | **Primer sequence (5’-3’)** | **Amplicon size** | **Reaction setup** | **Thermocycling conditions** | | **Reference** |
| --- | --- | --- | --- | --- | --- | --- | --- |
| *Leishmania* spp. | *cytB* | 1^st^ PCR | 919 bp | 25 µl reaction: 5 µl of DNA; 0.8 µM of each primer; 12.5 µl of NZYTaq 2 x Green Master Mix | 95 ºC - 3 min; 45 cycles [94 ºC - 1 min; 48 ºC - 1 min; 72 ºC - 1 min]; 72 ºC - 5 min | | [21] |
|  |  | Fw: AGCGGAGAGRARAGAAAAGG |  |  |  |  |  |
|  |  | Rev: CTACAATAAACAAATCATAATATRCAATT |  |  |  |  |  |
|  |  | 2^nd^ PCR | 568 bp | 25 µl reaction: 5 µl of 1^st^ PCR product^b^; 0.8 µM of each primer; 12.5 µl of NZYTaq 2 x Green Master Mix | 95 ºC - 3 min; 45 cycles [94 ºC - 1 min; 48 ºC - 1 min; 72 ºC - 1 min]; 72 ºC - 5 min | |  |
|  |  | Fw: GGTGTAGGTTTTAGTYTAGG |  |  |  |  |  |
|  |  | Rev: GYTCRCAATAAAATGCAAATC |  |  |  |  |  |
|  |  |  |  |  |  | |  |
|  | *g6pdh* | Fw: ATGTCGGAAGAGCAGTCT | 1689 bp | 25 µl reaction: 5 µl of DNA; 0.4 µM of each primer; 12.5 µl of NZYTaq 2 x Green Master Mix | 96 ºC - 5 min; 45 cycles [96 ºC - 1 min; 50 ºC - 1 min; 72 ºC - 90 s]; 72 ºC - 10 min | |  |
|  |  | Rev: TCACAGCTTATTCGAGGGAA |  |  |  |  |  |
|  |  |  |  |  |  |  |  |
|  | *hsp70* | 1^st^ PCR | 1286 bp | 25 µl reaction: 5 µl of DNA; 0.8 µM of each primer; 12.5 µl of NZYTaq 2 x Green Master Mix | 95 ºC - 5 min; 45 cycles [94 ºC - 40 seg; 61 ºC - 1 min; 72 ºC – 2 min]; 72 ºC - 10 min | |  |
|  |  | Fw: GGACGCCGGCACGATTKCT |  |  |  |  |  |
|  |  | Rev: CCTGGTTGTTGTTCAGCCACTC |  |  |  |  |  |
|  |  | 2^nd^ PCR | 741 bp | 25 µl reaction: 5 µl of 1^st^ PCR product^b^; 0.4 µM of each primer; 12.5 µl of NZYTaq 2 x Green Master Mix | 95 ºC - 5 min; 45 cycles [94 ºC - 40 s; 65 ºC - 1 min; 72 ºC - 1 min]; 72 ºC - 10 min | |  |
|  |  | Fw: GACAACCGCCTCGTCACGTTC |  |  |  |  |  |
|  |  | Rev: GTCGAACGTCACCTCGATCTGC |  |  |  |  |  |
|  |  |  |  |  |  |  |  |
|  | ITS rDNA | 1^st^ PCR | Variable | 25 µl reaction: 5 µl of DNA; 0.2 µM of each primer; 12.5 µl of NZYTaq 2 x Green Master Mix | 94 ºC - 3 min; 45 cycles [94 ºC - 30 s; 58 ºC - 30 s; 72 ºC - 90 s]; 72 ºC - 10 min | |  |
|  |  | Fw: GCTGTAGGTGAACCTGCAGCAGCTGGATCATT |  |  |  |  |  |
|  |  | Rev: GCGGGTAGTCCTGCCAAACACTCAGGTCTG |  |  |  |  |  |
|  |  | 2^nd^ PCR | Variable | 25 µl reaction: 5 µl of 1^st^ PCR product^b^; 0.2 µM of each primer; 12.5 µl of NZYTaq 2 x Green Master Mix | 94 ºC - 3 min; 45 cycles [94 ºC - 30 s; 58 ºC - 30 s; 72 ºC - 90 s]; 72 ºC - 10 min | |  |
|  |  | Fw: GCAGCTGGATCATTTTCC |  |  |  |  |  |
|  |  | Rev: AACACTCAGGTCTGTAAAC |  |  |  |  |  |
|  |  |  |  |  |  | |  |
|  | SSU rDNA | 1^st^ PCR | 603 bp | 30 µl reaction: 10 µl of DNA; 0.5 µM of each primer; 12.5 µl of NZYTaq 2 x Green Master Mix | 94 ºC - 5 min; 35 cycles [94 ºC - 30 s; 60 ºC - 30 s; 72 ºC - 30 s]; 72 ºC - 10 min | |  |
|  |  | Fw: GGTTCCTTTCCTGATTTACG |  |  |  |  |  |
|  |  | Rev: GGCCGGTAAAGGCCGAATAG |  |  |  |  |  |
|  |  | 2^nd^ PCR | 358 bp | 25 µl reaction: 5 µl of 1^st^ PCR product^c^; 0.6 µM of each primer; 12.5 µl of NZYTaq 2 x Green Master Mix | 94 ºC - 5 min; 32 cycles [94 ºC - 30 s; 65 ºC - 30 s; 72 ºC - 30 s]; 72 ºC - 10 min | |  |
|  |  | Fw: TCCATCGCAACCTCGGTT |  |  |  |  |  |
|  |  | Rev: AAAGCGGGCGCGGTGCTG |  |  |  |  |  |
|  |  |  |  |  |  | |  |
| *Felis catus* | *GUSB*^a^ | Fw: GCGTTCCTTTTGCGAGAGAG | 177 bp | 25 µl reaction: 5 µl of DNA; 0.4 µM of each primer; 12.5 µl of NZYTaq 2 x Green Master Mix | 95 ºC - 5 min; 30 cycles [95 ºC - 20 s; 55 ºC - 20 s; 72 ºC – 20 s]; 72 ºC - 7 min | |  |
|  |  | Rev: GCTGTGGAAGTTGCCCCTTA |  |  |  |  |  |

^a^PCR protocol designed in the course of this study.

^b^PCR product was previously diluted 1:50 in nuclease-free water.

^c^PCR product was previously diluted 1:200 in nuclease-free water.

Abbreviations: *cytB,* cytochrome *b*; g6pdh, glucose-6-phosphate dehydrogenase; *hsp70,* heat-shock protein 70; ITS rDNA, internal transcribed spacers-5.8 ribosomal DNA; SSU rDNA, small subunit ribosomal DNA; *GUSB*, beta-glucuronidase; bp, base pars.
